# Glutamine potentiates gentamicin to kill lab-evolved gentamicin-resistant and clinically isolated multidrug-resistant *Escherichia coli*

**DOI:** 10.3389/fmicb.2022.1071278

**Published:** 2022-12-02

**Authors:** Yue-tao Chen, Yan-mei Ma, Xuan-xian Peng, Hui Li

**Affiliations:** ^1^State Key Laboratory of Bio-Control, School of Life Sciences, Southern Marine Science and Engineering Guangdong Laboratory (Zhuhai), Guangdong Key Laboratory of Pharmaceutical Functional Genes, Sun Yat-sen University, Guangzhou, China; ^2^Laboratory for Marine Fisheries Science and Food Production Processes, Qingdao National Laboratory for Marine Science and Technology, Qingdao, China

**Keywords:** antibiotic resistance, glutamine, aminoglycoside, reprogramming metabolomics, multidrug resistance, *Escherichia coli*

## Abstract

**Introduction:**

Gentamicin is a conventional antibiotic in clinic. However, with the wide use of antibiotics, gentamicin-resistant *Escherichia coli* (E. coli) is an ever-increasing problem that causes infection in both humans and animals. Thus, it is especially important to restore gentamicin-mediated killing efficacy.

**Method:**

*E. coli* K12 BW25113 cells were passaged in medium with and without gentamicin and obtain gentamicin-resistant (K12-R_*GEN*_) and control (K12-S) strains, respectively. Then, the metabonomics of the two strains were analyzed by GC-MS approach.

**Results:**

K12-R_*GEN*_ metabolome was characterized as more decreased metabolites than increased metabolites. Meantime, in the most enriched metabolic pathways, almost all of the metabolites were depressed. Alanine, aspartate and glutamate metabolism and glutamine within the metabolic pathway were identified as the most key metabolic pathways and the most crucial biomarkers, respectively. Exogenous glutamine potentiated gentamicin-mediated killing efficacy in glutamine and gentamicin dose-and time-dependent manners in K12-R_*GEN*_. Further experiments showed that glutamine-enabled killing by gentamicin was effective to clinically isolated multidrug-resistant *E. coli*.

**Discussion:**

These results suggest that glutamine provides an ideal metabolic environment to restore gentamicin-mediated killing, which not only indicates that glutamine is a broad-spectrum antibiotic synergist, but also expands the range of metabolites that contribute to the bactericidal efficiency of aminoglycosides.

## Introduction

Conventional antibiotic treatments against bacterial infections are becoming ineffective due to the widespread antibiotic resistance worldwide, demanding the development of new antibiotics (Mühlberg et al., 2020; Vila et al., 2020). However, classical approaches that develop new antibiotics are not sufficient for the current pipeline, therefore new strategies are crucially needed to overcome antibiotic-resistant bacteria (Breijyeh et al., 2020; Gao et al., 2021; Ramanathan et al., 2021).

Recently, reprogramming metabolomics has been developed to effectively promote the bactericidal efficiency of existing antibiotics and restore anti-infective ability (Peng et al., 2015a; Cheng et al., 2019; Gong et al., 2020; Jiang et al., 2020, 2022; Yang et al., 2021a). Alanine, glucose, fructose, and glutamate reprogram an *Edwardsiella tarda* kanamycin-resistant metabolome into an *E. tarda* kanamycin-sensitive metabolome, which becomes susceptible to kanamycin-mediated killing (Peng et al., 2015b; Su et al., 2015, 2018). A similar effect has been determined in glutamine-reprogrammed multidrug-resistant *Escherichia coli*, glucose-reprogrammed gentamicin-resistant *Vibrio alginolyticus*, pyruvate-reprogrammed colistin-resistant *V. alginoliticus*, and nitrite- and glucose-reprogrammed *Pseudomonas aeruginosa*. Following the reprogramming, these antibiotic-resistant bacteria become sensitive to ampicillin-, gentamicin-, and colistin-mediated killing, respectively (Zhang et al., 2019, 2020; Li et al., 2020; Kuang et al., 2021, 2022; Zhao et al., 2021; Tang et al., 2022). Therefore, reprogramming metabolomics is a useful approach to combat antibiotic-resistant bacteria by using the existing antibiotics.

Aminoglycoside antibiotics are among the first antibiotics discovered and are one class of the existing antibiotics used. Among the class of antibiotics, gentamicin is a representative. Gentamicin is one of the most commonly used antibiotics worldwide because of its antimicrobial efficacy and the relatively low prevalence of clinical toxicity despite its toxicity to the kidney and the inner ear (Appel and Neu, 1978; Sha and Schacht, 1999). Especially, gentamicin is recommended as the empirical parenteral treatment for children with community-acquired urinary tract infections and as a crucial antibiotic for preventing orthopedic infections (Mosselhy et al., 2018; Roldan-Masedo et al., 2019). However, due to the widespread use of antibiotics, gentamicin-resistant *E. coli* is an ever-increasing problem that causes infection in both human health and animal feeding (Salas-Mera et al., 2017; Yamamoto et al., 2022). Therefore, restoration of gentamicin-mediated killing is highly demanded.

In this study, the reprogramming metabolomics approach was used to revert the resistance to gentamicin. First, *E. coli* K12 BW25113 cells were passaged in a medium with or without gentamicin to obtain a gentamicin-resistant strain (K12-R_*GEN*_) and a gentamicin-sensitive strain (K12-S), respectively. Then, gas chromatograph-mass spectromete (GC-MS) was used to investigate the metabolic profile of K12-R_*GEN*_ and identify glutamine as the most crucial biomarker. Finally, glutamine was shown to promote the gentamicin-mediated killing efficiency to both lab-evolved K12-R_*GEN*_ and clinically isolated multidrug-resistant *E. coli*.

## Materials and methods

### Bacterial strains used

In the present study, *E. coli* K12 BW25113 [genotype, Δ(*araD-araB*)567, Δ*lacZ*4787(:*rrnB*-3), *lambda*-, *rph-1*, Δ(*rhaD-rhaB*)568, *hsdR*514] was taken from the KEIO collection. A single colony of *E. coli* K12 BW25113 was picked from the Luria-Bertani (LB) agar plate and cultured in LB medium for 16 h at 37°C. The overnight cultures were diluted 1:100 in fresh LB medium and grew to phase OD_600_ of 0.5 at 37°C. These bacteria were passaged in LB medium with and without gentamicin for gentamicin-resistant strains and control, respectively. The three strains, ancestor strain (K12), gentamicin-resistant strain (K12-R_*GEN*_), and control strain (K12-S), were collected to determine the minimum inhibitory concentration (MIC) by antimicrobial susceptibility testing.

### Minimum inhibitory concentration measurement

Measurement of MIC was performed as previously described (Kuang et al., 2021). In brief, 160 μg/ml of gentamicin sulfate [Sangon Biotech (Shanghai) Co., Ltd.] was serially double diluted by row in a 96-microwell plate. The overnight culture was diluted at 1:100 into 5 ml LB medium and grew to a phase of 0.5 at OD_600_. A bacterial sample of 5 × 10^4^ CFU was then added to each well. After incubating for 16 h at 37°C, the bacteria growth in each well was recorded. The antibiotic concentration of a well without bacteria growth is the MIC of the tested strain. Data were obtained from three biological replicates.

### Growth curve analysis

The overnight cultures were diluted 1:100 in LB medium and grew at 200 rpm at 37°C. Then, the optical density of the culture at OD_600_ was measured every 2 h. The growth curve was drawn using GraphPad Prism version 8.0. At least three biological replicates were performed.

### Survival capability assay

The overnight culture was diluted 1:1000 into tubes with 5 ml LB medium. The tube was added to gentamicin with different concentrations. After growing for 6 h at 200 rpm at 37°C, the optical density of the culture at OD_600_ was measured. The survival percentage was calculated as follows: optical density of the culture with different concentrations of antibiotic divided by that without antibiotic.

### Metabolomics analysis

#### Metabolic profiling

Sample preparation was carried out according to the previously reported study (Su et al., 2018). In brief, the overnight cultured were diluted 1:100 into 50 ml LB broth. After incubating for about 4 h until a growth phase of 1.0 at OD_600_ nm, bacterial metabolism was quenched by adding a 2-fold volume of ice-cold methanol. Cells were collected, resuspended, and adjusted into an OD_600_ of 1.0 with PBS. A total of 10 ml of suspension were collected, and the pellet was added into 1 ml pre-cooled methanol (HPLC grade) immediately. Subsequently, 10 μl of 0.1 mg/ml of ribitol (Sigma) was added as an internal quantitative standard. Intracellular metabolites were extracted by ultrasonic crushing, and the supernatant was evaporated by a vacuum centrifuge dryer (Labconco, USA). For derivatization, 80 μl of methoxyamine hydrochloride (20 mg/ml in pyridine) was added to each dried sample and incubated for 3 h at 37°C. Subsequently, 80 μl of N-methyl-N-(trimethylsilyl) trifluoroacetamide (MSTFA, Sigma) was added and incubated for 45 min at 37°C. Metabolites were analyzed by GC-MS using an Agilent 7890A GC and 5975C VL MSD quadrupole MS (Agilent Technologies, USA).

#### Gas chromatograph-mass spectromete data analysis

The statistical analysis was performed as described previously (Kuang et al., 2021). In brief, compounds were tentatively identified by matching their retention time and mass spectra with structures available in the NIST library in the Xcalibur software (version 2.1). The peak area corresponding to each metabolite was normalized based on the Ribitol (internal standard) and total peak area in the sample. Subsequently, metabolites were scaled by the quartile range in the sample. The Mann–Whitney U-test (α = 0.05) with SPSS statistics 17.0 (IBM, USA) was used to compare the difference in abundance of metabolites between the two groups. The R software (R × 64 4.0.3) was used for cluster analysis. Principle component analysis and S-plot analysis were conducted using SIMCA-P + (Version 12.0) software. Enriched metabolic pathways were identified using the MetaboAnalyst online website.^1^ Data were plotted using GraphPad Prism version 8.0.

### Bactericidal assay

Overnight bacterial cultures were collected by centrifugation at 8,000 rpm for 3 min and washed three times with sterile saline. To confirm the drug resistance of K12-R_*GEN*_, precipitates were adjusted to OD_600_ of 0.2 and then diluted 100-fold using a fresh LB medium. K12-S was used as a control. To investigate whether glutamine improved the sensitivity of bacteria to antibiotics, precipitates were adjusted to OD_600_ of 0.2 and then diluted 100-fold using M9 minimal medium with 10 mM NaAc, 2 mM MgSO_4_, and 0.1 mM CaCl_2_. Each tube was added to 5 ml of diluted bacterial solution in the presence and/or absence of gentamicin and glutamine. After growing for 6 h with 200 rpm at 37°C, 100 μl of cultures were serially 10-fold diluted and 5 μl of cultures were plated onto LB agar. Only the clearly visible colonies were counted and multiplied by the dilution. Percent survival was determined by dividing the CFU obtained from a treated sample by those from the control.

## Results

### K12-R_*GEN*_ exhibit resistance characteristics

**FIGURE 1:**
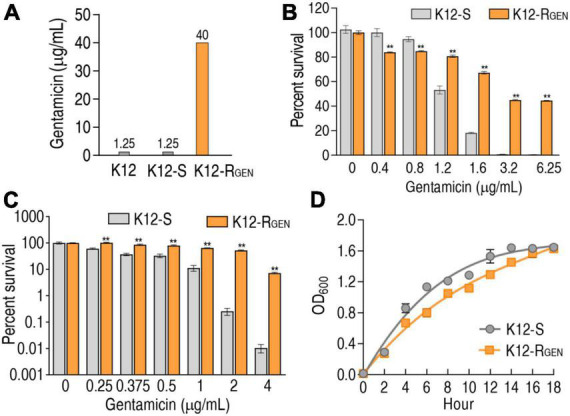
Antibiotic resistance phenotypes of K12-R_GEN_. **(A)** MIC of K12-R_GEN_. **(B)** Survival of K12-R_GEN_ to a lethal dose of gentamicin. **(C)** Survival capability of K12-R_GEN_ to a non-lethal dose of gentamicin. **(D)** Growth curve of K12-R_GEN_. Results are displayed as mean ± SEM and three biological repeats are performed. Significant differences are identified. **p < 0.01.

K12 was passaged in LB medium with or without 1/2 minimum inhibitory concentration (MIC) and became K12-R_*GEN*_ and K12-S, respectively. MIC of the three strains was measured using a microplate method. The passage led to 32 MIC (40 μg gentamicin) of K12-R_*GEN*_ and 1 MIC (1.25 μg gentamicin) of K12-S compared with their parent strain (Figure 1A), suggesting that K12-R_*GEN*_ was a gentamicin-resistant strain. To further demonstrate the resistance, survival capability and bactericide assays were performed. The survival capability of the two strains was reduced with increasing gentamicin concentration, but higher survival was detected in K12-R_*GEN*_ than in K12-S (Figure 1B). Equally, higher viability was found in K12-R_*GEN*_ than in K12-S in the bactericide assay (Figure 1C). Finally, the growth curve showed slower growth in K12-R_*GEN*_ than in K12-S (Figure 1D). These results indicate that K12-R_*GEN*_ is a gentamicin-resistant strain with a differential-resistant phenotype.

### Gentamicin-mediated resistant metabolome

**FIGURE 2:**
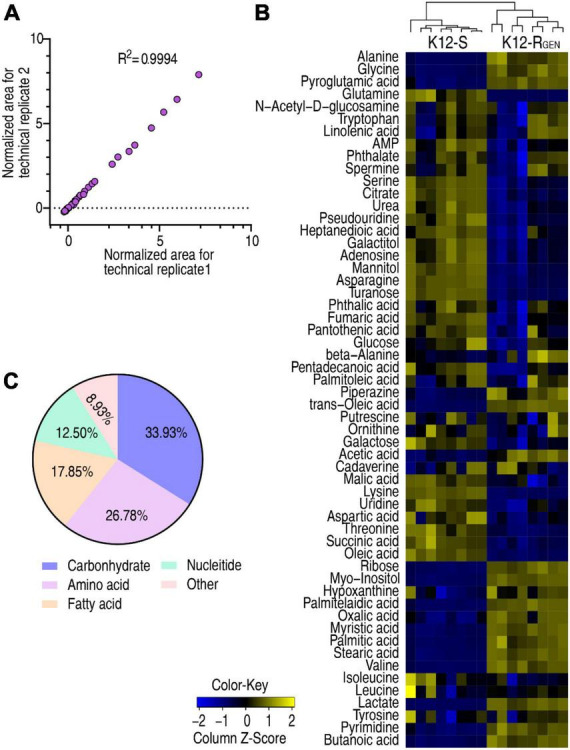
Metabolite profiling of K12-R_GEN_ and K12-S**. (A)** Reproducibility of the metabolomic profiling platform used in the discovery phase. The abundance of metabolites quantified in samples over two technical replicates is shown. The Pearson correlation coefficient between technical replicates varies between 0.9946 and 0.9995. **(B)** Heat map of unsupervised hierarchical clustering of different metabolites (row). Blue indicates decreases and yellow indicates an increase of the metabolites scaled to the mean and standard deviation of row metabolite level (see color scale). **(C)** Categories of the differential metabolites. Fifty-six differential abundances of metabolites are searched against in KEGG for categories. The pie chart is generated in Excel 2010 (Microsoft, USA).

To understand metabolic alterations related to the resistance, a GC-MS-based metabolomics approach was used to characterize the metabolic profile of K12-R_*GEN*_ compared with K12-S. Four biological samples with two technical repeats in each group yielded 16 data sets. The correlation coefficient between technical replicates varied between 0.9946 and 0.9995, demonstrating the reproducibility of the data (Figure 2A). A total of 240 aligned individual peaks were obtained from each sample. After the removal of internal standard ribitol and any known artificial peaks, 56 metabolites were identified as shown in Figure 2B. Among them, 33.93%, 26.78%, 17.85%, 12.50%, and 8.93% were categorized as carbohydrates, amino acids, fatty acids, nucleotides, and others, respectively (Figure 2C). These results indicate that K12-R_*GEN*_ has a metabolome that is different from that of K12-S.

### Gentamicin-mediated differentially resistant metabolome

**FIGURE 3:**
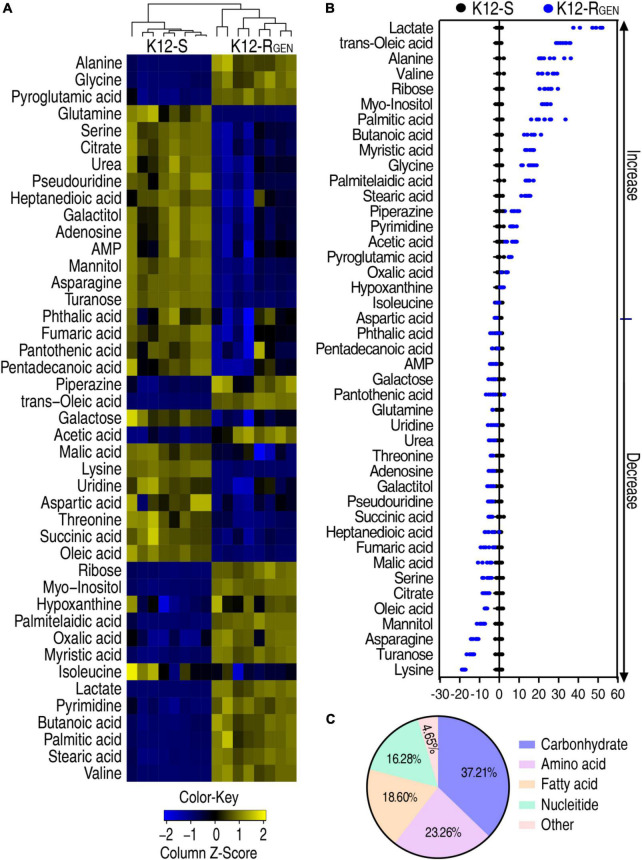
Differential metabolic profiling between K12-R_GEN_ and K12-S. **(A)** Heat map showing the differential abundance of metabolites. Yellow and blue indicate an increase and decrease of metabolites relative to the median metabolite level of the control, respectively (see color scale). **(B)** A Z-score plot of differential metabolites based on control. Each point represents one metabolite in one technical repeat and is colored by sample types. **(C)** Category of these differential abundances of metabolites.

To gain a differential abundance of metabolites between K12-R_*GEN*_ and K12-S, a two-sided Mann–Whitney U-test coupled with a permutation test was utilized. Using the analysis, a total of 43 differential abundance of metabolites were identified in K12-R_*GEN*_ (Figure 3A). The Z-value showed the dispersion of data with 18 upregulation and 25 downregulation (Figure 3B). These differential abundances of metabolites were classified into five categories. Among them, 37.21%, 23.26%, 18.60%, 16.28%, and 4.65% belonged to carbohydrates, amino acids, nucleotides, lipids, and others, respectively (Figure 3C). Therefore, a metabolic shift was determined in K12-R_*GEN*_.

### Gentamicin-mediated enriched metabolic pathways

**FIGURE 4:**
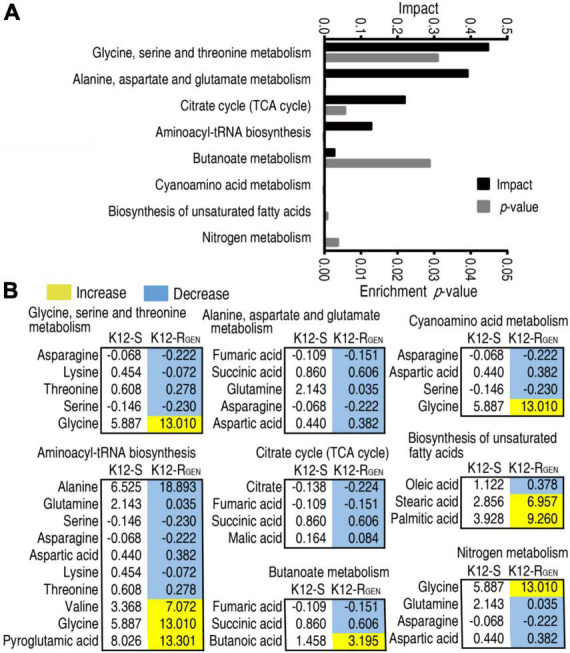
Pathway enrichment analysis. **(A)** Pathway enrichment of differential metabolites in K12-R_GEN_. **(B)** Integrative analysis of metabolites in significantly enriched pathways. Yellow and light blue indicate increased and decreased metabolites, respectively.

A metabolic pathway is a set of biochemical reactions that the cells need to carry out their function. Thus, it is especially important to know the metabolic pathways enriched by these differential abundances of metabolites for understanding gentamicin-mediated metabolic alteration. Metabolic pathway enrichment analysis showed that eight metabolic pathways were enriched. According to the impact, they were ranked from high to low as follows: glycine, serine, and threonine metabolism > alanine, aspartate, and glutamate metabolism > TCA cycle > aminoacyl-tRNA biosynthesis > butanoate metabolism > cyanoamino acid metabolism > biosynthesis of unsaturated fatty acids > nitrogen metabolism (Figure 4A). Integrative analysis showed that among the eight enriched metabolic pathways, all metabolites of alanine, aspartate, and glutamate metabolism and TCA cycle were decreased (Figure 4B). These findings with the above more depressed metabolites than elevated metabolites in the gentamicin-mediated metabolome together suggest that the depressed metabolic pathway plays a key role in the resistance. Meanwhile, alanine, aspartate, and glutamate metabolism fuel the TCA cycle. Thus, alanine, aspartate, and glutamate metabolism can be identified as the most important metabolic pathways.

### Gentamicin-mediated biomarkers

**FIGURE 5:**
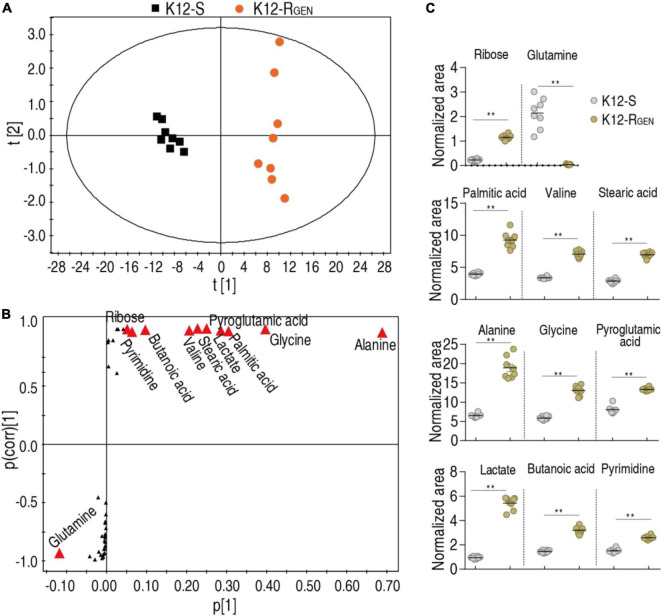
Identification of crucial metabolites. **(A)** PCA analysis according to the treatments set. Each dot represents the technical replicate analysis of samples in the plot. **(B)** S-plot generates from OPLS-DA. Predictive component p [1] and correlation p(corr) [1] differentiate K12-R_GEN_ from K12-S. The dot represents metabolites and candidate biomarkers are highlighted in red. **(C)** Scatter plot of biomarkers in data **(B)**. Results **(C)** are displayed as mean ± SEM, and significant differences are identified (**p < 0.01) as determined by a two-tailed Student’s *t*-test.

Biomarker(s) may provide a differential metabolome value and thereby identification of biomarkers is a key step in the analysis of metabolomics. Thus, orthogonal partial least square-discriminate analysis (OPLS-DA) was conducted to recognize the sample pattern. Component t [1] differentiated K12-R_*GEN*_ from K12-S and Component t [2] discriminated variation within the two groups (Figure 5A). Discriminating variables were displayed with an S-plot when we set cutoff values as greater or equal to 0.05 and 0.5 for the absolute value of covariance p and correlation p(corr), respectively. Among these metabolites used for the analysis, 11 played more roles than the others in the differentiation and were identified as biomarkers (Figure 5B). The scatter plot showed their differential abundances between K12-R_*GEN*_ and K12-S, where only glutamine was depressed in K12-R_*GEN*_ (Figure 5C). Glutamine belongs to alanine, aspartate, and glutamate metabolism. Reports have shown that the complementation of crucially depressed metabolites may restore antibiotic-mediated killing efficacy (Peng et al., 2015b; Zhao et al., 2021). Therefore, glutamine as the crucial biomarker may revert the resistance.

### Glutamine-potentiated gentamicin-mediated killing

**FIGURE 6:**
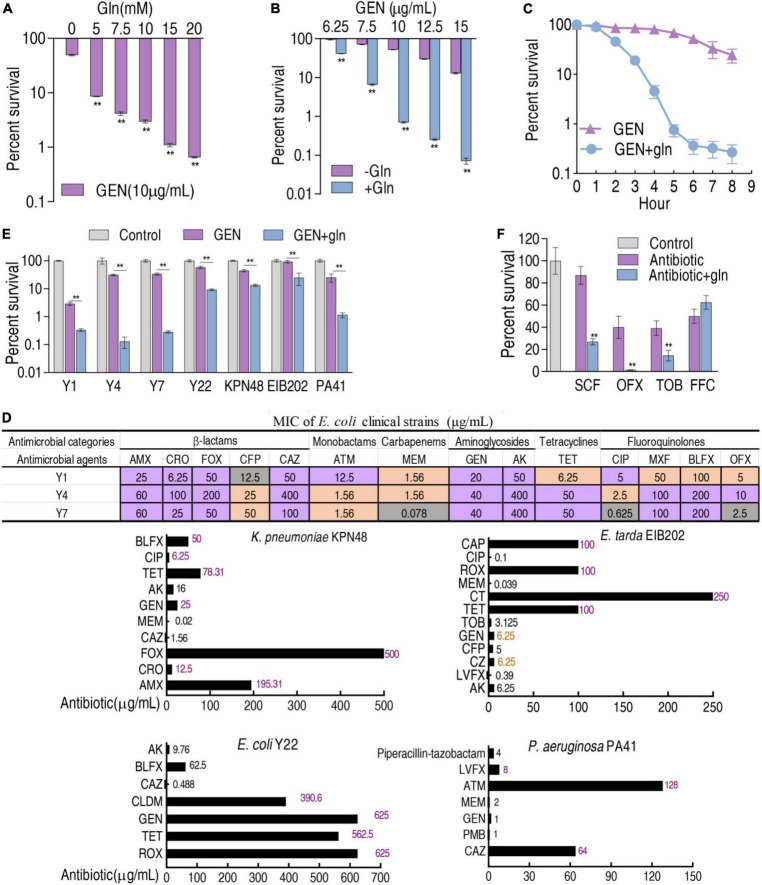
Glutamine promotes gentamicin-mediated killing. **(A)** Percent survival of K12-R_GEN_ in the presence of the indicated concentration of glutamine and 10 μg gentamicin. **(B)** Percent survival of K12-R_GEN_ in the presence of the indicated concentration of gentamicin and with or without 20 mM glutamine. **(C)** Percent survival of K12-R_GEN_ in the indicated incubation time plus 20 mM glutamine and 10 μg gentamicin. The concentration of K12-R_GEN_ in **(A–C)** was 5 × 10^8^ CFU/ml. **(D)** MIC measurement of clinically isolated bacterial strains in four to six kinds of antibiotics commonly used in clinical practice. Purple indicates resistant; orange indicates intermediate; dark gray indicates susceptible. For AMX, CRO, FOX, CFP, CAZ, MEM, GEN, CIP, TET, CLDM, PMB, LVFX, CZ, CT, TOB, CAP, and ROX, the standard was according to reference (CLSI, 2012). For ATM, OFX, and AK, the standard was according to reference (Kahlmeter et al., 2006). For MXF and BLFX, susceptible, intermediate, and resistant *E. coli* were defined as MIC ≤ 0.025, MIC = 0.05, and MIC ≥ 0.1 and MIC ≤ 0.05, MIC = 0.1, and MIC ≥ 0.2, respectively. **(E)** Percent survival of clinically isolated strains in the presence or absence of gentamicin (Y1 at 2 μg/ml; Y4, Y7 at 2.5 μg/ml; Y22 at 100 μg/ml; *K. pneumoniae* KPN48 (2 μg/ml); *E. tarda* EIB202 (2 μg/ml); *P. aeruginosa* PA41 (1 μg/ml), or in the presence of both gentamicin and 20 mM glutamine. **(F)** Percent survival of K12-R_GEN_ in the indicated antibiotics (SCF, 10 μg/ml; OFX, 1.5 μg/ml; TOB, 2.5 μg/ml; FFC, 40 μg/ml) with and without 20 mM glutamine. The concentration of clinically isolated strains in **(E)** and K12-R_GEN_ in **(F)** was 1 × 10^6^ CFU/ml. Amoxicillin (AMX), Ceftriaxone (CRO), Cefoxitin (FOX), Cefoperazone (CFP), Cefoperazone-sulbactam (SCF), Ceftazidime (CAZ), Aztreonam (ATM), Meropenem (MEM), Gentamicin (GEN), Amikacin (AK), Ciprofloxacin (CIP), Moxifloxacin (MXF), Balofloxacin (BLFX), Ofloxacin (OFX), Tetracycline (TET), Clindamycin (CLDM), Polymyxin B (PMB), Levofloxacin (LVFX), Cefazolin (CZ), Colistin (CT), Tobramycin (TOB), Chloramphenicol (CAP), Roxithromycin (ROX), Florfenicol (FFC). Results are displayed as mean ± SEM and three biological repeats are performed. Significant differences are identified. **p < 0.01.

To test whether glutamine reverted gentamicin resistance to increase bacterial sensitivity to gentamicin, gentamicin and glutamine were synergistically used to kill K12-R_*GEN*_. Glutamine promoted gentamicin-mediated killing in a dose-dependent manner (Figure 6A). When 20 mM glutamine was used, the killing efficacy was elevated with increasing gentamicin dose (Figure 6B). The killing efficacy was also incubation period-dependent (Figure 6C). Therefore, glutamine-potentiated gentamicin-mediated killing is effective for lab-evolved gentamicin-resistant *E. coli*. On the other hand, four clinically isolated multidrug-resistant *E. coli* strains and three clinically isolated multidrug-resistant bacteria were used to test the glutamine-induced potentiation (Figure 6D). Lower survival was detected in the synergistic use of gentamicin and glutamine than in gentamicin alone (Figure 6E). Therefore, the glutamine-potentiated gentamicin-mediated killing is effective for both lab-evolved gentamicin-resistant and clinically isolated multidrug-resistant *E. coli*. Furthermore, lower survival was also detected in the synergistic use of glutamine and other antibiotics, such as cefoperazone-sulbactam, ofloxacin, and tobramycin than antibiotic alone (Figure 6F).

## Discussion

Metabolic environments confound antibiotic-mediated killing (Lee and Collins, 2011; Kuang et al., 2021; Zhao et al., 2021; Su et al., 2022; Tang et al., 2022). However, information regarding the metabolites-enabled killing of *E. coli* by gentamicin is not available. The present study explores how to provide a metabolic environment that potentiates gentamicin-mediated killing efficacy. To do this, the metabolic profile of lab-evolved *E. coli* K12-R_GEN_ is compared with that of control K12-S. The comparison shows that K12-R_GEN_ has a gentamicin-resistant metabolome, characterizing more decreased metabolites than increased metabolites and depression of all or almost metabolites in most enriched metabolic pathways. Glutamine and alanine and aspartate and glutamate metabolisms are identified as the most crucial biomarkers and the most key metabolic pathways, respectively. Exogenous glutamine-potentiated reverting causes K12-R_GEN_ and clinically isolated multidrug-resistant *E. coli* to be sensitive to gentamicin. Therefore, glutamine provides an ideal metabolic environment to restore gentamicin-mediated killing.

Metabolites-enabled killing efficacy by antibiotics is related to both antibiotic types and classes and bacterial species (Peng et al., 2015b; Zhao et al., 2021). Although glutamine potentiates antibiotic-mediated killing has been reported, only glutamine-enabled killing of *E. coli* by ampicillin, of *Salmonella* by apramycin, and of *Mycobacterium* persisters by rifampicin are carefully studied (Huang et al., 2018; Yong et al., 2021; Zhao et al., 2021). The present study identifies metabolites that potentiate gentamicin-mediated killing efficacy and determines glutamine-enabled killing of lab-evolved gentamicin-resistant *E. coli* and clinically isolated multidrug-resistant *E. coli.* This finding not only supports the conclusion that glutamine is a broad-spectrum antibiotic synergist but also provides an ideal way by which gentamicin-mediated killing is restored.

The metabolites-enabled killing of bacteria by aminoglycoside antibiotics including kanamycin and gentamicin has been investigated (Allison et al., 2011; Peng et al., 2015b). Allison et al. show glucose-enabled eradication of bacterial persisters (Allison et al., 2011). Peng et al. (2015b) and Su et al. (2015) utilize alanine, glucose, and fructose to reprogram kanamycin-resistant and multidrug-resistant *Edwardsiella tarda* metabolomes into sensitive metabolomes and thereby lead to the elevation of kanamycin-mediated killing efficacy. Zhang et al. demonstrate glucose-enabled killing of antibiotic-resistant *Vibrio alginolyticus* by gentamicin based on reprogramming metabolomics (Zhang et al., 2019, 2020). In addition, Lv et al. (2022) find that a non-metabolite, n-butanol, also potentiates aminoglycosides-mediated killing efficacy. The present study exhibits glutamine-enabled killing of lab-evolved gentamicin-resistant *E. coli* and clinically isolated multidrug-resistant *E. coli* by gentamicin. This finding expands the range of metabolites that contribute to the bactericidal efficiency of aminoglycosides.

Notably, more studies on metabolomics-related antibiotic resistance are carried out by using lab-evolved antibiotic-resistant strains or clinically isolated antibiotic-resistant pathogens (Li et al., 2018; Wen et al., 2021; Guan et al., 2022). The present study utilizes a lab-evolved antibiotic-resistant strain to identify a metabolite that provides a metabolic environment for restoring antibiotic-mediated killing and then to demonstrate the efficacy of the metabolite in eliminating clinically isolated multidrug-resistant pathogenic *E. coli*. Thus, this approach is effective in identifying metabolites-enabled killing of clinically isolated bacteria by antibiotics.

## Conclusion

A metabolome-reprogramming approach, which has been demonstrated to be effective in reverting resistance and restoring anti-infective ability (Peng et al., 2015a; Gong et al., 2020; Jiang et al., 2020, 2022; Yang et al., 2021b), is used to understand gentamicin-resistant metabolic mechanisms. This leads to the identification of depressed glutamine and inactivated alanine and aspartate and glutamate metabolism as the most crucial biomarkers and the most key metabolic pathways, respectively. Exogenous glutamine reverts gentamicin resistance of lab-evolved gentamicin-resistant and clinically isolated multidrug-resistant *E. coli*. These results provide a solid foundation for further preclinical research.

## Data availability statement

The original contributions presented in this study are included in the article/supplementary material, further inquiries can be directed to the corresponding author.

## Author contributions

HL conceptualized the project and designed the protocol. Y-TC and Y-MM performed the experiments and interpreted the data. HL and X-XP wrote the manuscript. All authors contributed to the article and approved the submitted version.

## Footnotes

1
https://www.metaboanalyst.ca/
